# Impact of prosthesis–patient mismatch on short-term outcomes after aortic valve replacement: a retrospective analysis in East China

**DOI:** 10.1186/s13019-017-0596-2

**Published:** 2017-05-25

**Authors:** Lei Guo, Junnan Zheng, Liangwei Chen, Renyuan Li, Liang Ma, Yiming Ni, Haige Zhao

**Affiliations:** 1 Department of Thoracic and Cardiovascular Surgery, First Affiliated Hospital of Zhejiang University, School of Medicine, Hangzhou, China; 2 Department of Cardiothoracic Surgery, the First Affiliated Hospital, Zhejiang University, 79 Qingchun Road, Hangzhou, 310003 China

**Keywords:** Prosthesis–patient mismatch (PPM), Aortic valve replacement (AVR), Aortic stenosis (AS), Effective orifice area (EOA)

## Abstract

**Background:**

Prosthesis–patient mismatch (PPM) may affect the clinical outcomes of patients undergoing aortic valve replacement (AVR). We aimed to determine the incidence of PPM, its effect on short-term mortality, and the factors contributing to PPM in China.

**Methods:**

We retrospectively examined all consecutive patients with isolated or concomitant AVR at our hospital between January 1, 2013 and December 31, 2015. PPM was defined as an effective orifice area index (EOAi) of ≤ 0.85 cm^2^/m^2^. The baseline, echocardiographic, operative, and outcome data of all patients were collected from the national database.

**Results:**

A total of 869 patients were included in the study. PPM was detected in 15.9% (138/869) of the patients. Four patients (0.5%) met the criteria for severe PPM. Patients with PPM were older and had a higher prevalence of diabetes, coronary heart disease, aortic stenosis (AS), and preoperative left ventricular dysfunction but a lower incidence of smoking history and aortic regurgitation. Logistic regression analysis showed that female gender (*P* < 0.001), AS (*P* = 0.014), higher body mass index (BMI) (*P* < 0.001), and bioprosthesis (*P* < 0.001) were independent predictors of PPM. We also found that PPM (*P* = 0.005) was associated with 30-day all-cause mortality, along with smoking history (*P* = 0.001) and low preoperative left ventricular ejection fraction (LVEF) (*P* = 0.004).

**Conclusions:**

PPM is associated with high short-term mortality after AVR in China. Female gender, aortic stenosis, bioprosthesis, and high BMI are risk factors for the incidence of PPM.

## Background

Prosthesis–patient mismatch (PPM) after aortic valve replacement (AVR) surgery was first mentioned in 1978 by Rahimtoola, and has been a topic of discussion ever since [1]. PPM occurs when the effective orifice area (EOA) of the implanted prosthesis is too small in relation to the patient’s body size.

The clinical significance of PPM after AVR remains controversial even after 37 years since its first description. Some studies have showed favorable results despite the occurrence of PPM after AVR [2–4], while several other clinical studies have demonstrated that aortic PPM might be associated with an increased incidence of long- and short-term adverse outcomes, including cardiac-related death [5, 6]. Patients with valvular diseases in East China have smaller body surface area, more rheumatic causes and lower anticoagulation intensity requirements [7]. Therefore the incidence, predictions and complications of PPM in East China patients might differ greatly from patients in the western countries. However, study regarding PPM in this specific group of patients has seldom been reported.

For these reasons, we retrospectively analyzed PPM in patients undergoing first time isolated or concomitant AVR in East China and aimed to determine the incidence of PPM, its effect on short-term mortality, and the factors contributing to PPM.

## Methods

### Patient population and data collection

This study was conducted at a single large cardiothoracic surgical center in southeast China. After obtaining written informed consent waived by the Hospital Review Board, we reviewed data from the Chinese Adult Cardiovascular Surgery Database, which holds clinical information on all patients at the center, the Department of Cardiothoracic Surgery at the First Affiliated Hospital, Zhejiang University, China, since April 2013.

We analyzed all consecutive patients aged > 18 years undergoing first time isolated or concomitant AVR at the center from January 1^st^, 2013 to December 31^st^, 2015. Patients requiring a composite valve vascular prosthesis procedure were excluded.

In total, 869 patients were included in this study. Baseline, operative, and outcome data of the patients were prospectively collected, validated, and entered into the database, which was queried retrospectively. A 30-day postoperative follow-up was conducted for all discharged patients at the outpatient clinic.

### PPM and definitions

Body surface area (BSA) was derived from the Dubois formula. The aortic valve prosthesis effective valve orifice area (EOA) was derived from *in vitro* measurements provided by the manufacturers and from scientific publications, as outlined in Table 1.

**Table 1:** *In vivo* effective orifice area values (cm^2^) corresponding to each valve

Valve prosthesis	No. of patients n(%)	17 mm 1.8%	19 mm 8.7%	21 mm 27.7%	23 mm 30.0%	25 mm 22.6%	27 mm 7.0%	29 mm 1.5%	31 mm 0.6%	ref
Mechanical										
CarboMedics Orbis Universal Valve	517(59.5)	1	1.2	1.5	1.7	2	2.5	2.6	2.7	[19]
ATS Open Pivot 500FA (size, mm)	10(1.2)	0.6 (16)	1	1.6	1.8	2.2	2.5	3.1		[15]
ATS Open Pivot AP360 (size, mm)	2(0.2)	1.2 (16)	1.5 (18)	1.7 (20)	2.1 (22)	2.5 (24)	3.1 (26)			[20]
St. Jude Regent Valve	40(4.6)	1.2	1.6	2	2.2	2.5	3.6	4.4		[21]
St. Jude Master Series	90(10.4)	1	1.2	1.5	2.0	2.6	3.1			[22]
Bioprosthesis										
Hancock II Procine Bioprosthesis	120(13.8)			1.2	1.3	1.5	1.6	1.6		[19]
Mosaic Procine Bioprosthetic Valve	4(0.5)		1.1	1.2	1.4	1.7	1.8	2		[23]
St. Jude Biocor Stented Tissue Valve	47(5.4)			1.3	1.6	1.8	2			[24]
Carpentier-Ed PERIMOUNT	32(3.7)		1.1	1.3	1.5	1.8	2.1	2.2		[19]
Carpentier-Ed PERIMOUNT MAGNA	7(0.8)		1.3	1.7	2.1	2.3				[24]
PPM rate of each valve sizes		81.2%	52.6%	19.1%	10.7%	4.6%	3.3%	0%	0%	

*PPM* prosthesis patient mismatch, *ref* reference

EOA was divided by BSA to obtain the effective orifice area index (EOAi). PPM was defined as EOAi ≤ 0.85 cm^2^/m^2^ [8]. EOAi ≤ 0.65 cm^2^/m^2^ was considered severe PPM.

Chronic renal insufficiency: serum creatinine ≥ 2 mg/dl. Peripheral arterial disease: claudication, carotid stenosis > 50% or previous/planned intervention on the abdominal aorta, limb arteries or carotids. Coronary artery disease: ≥ 50% reduction in one or more coronary vessels in single or more plane angiographic images. Emergency surgery: operation required within 24 h of onset of symptoms. Postoperative renal failure: the increase in baseline creatinine greater than 2 mg/dl.

### Surgical technique

The surgical records of all patients were reviewed. A total of 210 aortic valve bioprostheses and 659 aortic valve mechanical prostheses were implanted. The prostheses used included: Hancock II Procine Bioprosthesis (120 patients) and Mosaic Procine Bioprosthetic Valves (four patients) (Medtronic, Inc, Minneapolis, Minn), Biocor Stented Tissue Valve (47 patients) (St Jude Medical, Inc, St Paul, Minn), Carpentier-Edwards Perimount (32 patients) and Carpentier-Edwards Perimount Magna Ease (seven patients) (Baxter Healthcare Corp, Edwards Division, Santa Ana, Calif), CarboMedics Orbis Universal Valve (517 patients) (CarboMedics, Inc, Austin, TX), ATS Open Pivot 500FA, 500DM (ten patients) and ATS Open Pivot AP360 (two patients) (ATS Medical, Inc, Minneapolis, Minn), St Jude Regent Valve (40 patients) and St Jude Master Series Heart Valves (90 patients) (St Jude Medical, Inc, St Paul, Minn) (Table 1).

An isolated or concomitant aortic valve replacement was performed in all patients. Concomitant operations in some of the patients included coronary artery bypass grafting and other heart valve procedures. Standard anesthesia and surgical technique, extracorporeal circulation and myocardial protection methods were used. Most of the patients were approached through a full median sternotomy followed by antegrade 4:1 cold blood cardioplegia for myocardial protection. Antegrade plus retrograde cardioplegia was applied for patientis with coronary stenosis. Intermittent perfusion of cold blood cardioplegia was maintained, with a frequency of once every 20 min.

After consulting the patients and their relatives preoperatively, a decision on the type of prosthesis was made by the surgeon, taking into consideration the preoperative characteristics and the intraoperative findings. The largest suitable prosthesis was chosen. When performing the valve replacement procedure, we applied simple interrupted mattress suturing with pledget on the left ventricular side of the annulus to ensure a larger orifice area and a shorter cross-clamp time.

### Statistical analysis

The Kolmogorov-Smirnov test and/or Shapiro-Wilk test was used to verify the normality of the quantitative variables as appropriate. Continuous variables were presented as mean ± standard deviation, whether Gaussian distributed or median (interquartile range). Categorical variables were expressed as an absolute number (percentage). Pearson’s χ2 test was used for descriptive, univariate statistics, such as the comparison of portions, while Student’s unpaired T-test was used for normally distributed data comparisons. The Mann-Whitney U test was used for group comparison of continuous non-Gaussian distributed variables. Two-tailed *P*-values were derived from the calculated test statistics, and *P* ≤ 0.05 was considered statistically significant. Binary multivariate logistic regression analysis by the forward method was performed to study the factors affecting PPM and mortality. SPSS software for windows (version 19.0) was used to analyze the data.

## Results

### Patient characteristics and preoperative data

A total of 869 consecutive patients were included in the study. PPM was detected in 15.9% (138/869) of the patients. Four patients (0.5%) met the criteria for severe PPM. Smaller valve sizes are related to higher rate of PPM (Table 1). Compared with the non-PPM group, patients with PPM were older and had a higher prevalence of diabetes, coronary heart disease, AS, and preoperative left ventricular ejection fraction (LVEF). But the incidence of smoking history and aortic regurgitation was relatively low in PPM patients (Table 2).

**Table 2:** Preoperative patient characteristics

Preoperative data	Non-PPM (*n* = 731)	PPM (*n* = 138)	*P* value
Age, y	56(47–62)	64(55–69)	<0.001
Male	394(54%)	57(41%)	0.07
BMI(kg/m2)	22.58 ± 3.29	23.51 ± 5.92	0.076
BSA(m2)	1.61 ± 0.17	1.60 ± 0.14	0.484
Smoking history	138(18.9%)	14(10.1%)	0.014
Diabetes	63(8.6%)	22(15.9%)	0.008
Hypertension	178(24.4%)	39(28.1%)	0.336
Chronic renal insufficiency	23(3.1%)	5(3.6%)	0.77
Peripheral arterial disease	21(2.9%)	5(3.6%)	0.64
Cerebrovascular accident:	15(2.1%)	2(1.1%)	0.99
Coronary heart disease	78 (10.7%)	24(17.4%)	0.024
NYHA functional class (≥ III)	260(35.6%)	51(37.1%)	0.772
AF	212(29.0%)	42(30.1%)	0.76
Previous myocardial infarction	1(0.1%)	1(1.1%)	0.293
AS	399(55.0%)	90(65.1%)	0.031
Aortic regurgitation (moderate to severe)	508(69.5%)	79(57.1%)	0.006
LVEF	61.02 ± 8.99	62.00 ± 9.18	0.105
Emergency surgery	3(0.4%)	0(0.0%)	0.99
Aspirin within 5 days before surgery	5(0.7%)	1(1.1%)	0.99
Clopidogrel within 5 days before surgery	1(0.1%)	1(1.1%)	0.293

*PPM* prosthesis-patient mismatch; *BMI* body mass index; *BSA* body surface area; *NYHA* New York Heart Association; *AF* atrial fibrillation; *AS* aortic valve stenosis; *LVEF* left ventricular ejection fraction

### Operative data

As shown in Table 3, there were no significant differences between the groups regarding cardiopulmonary bypass (CPB) time, aortic cross-clamp time, and mitral valve procedure combined. However, remarkably more patients with prosthesis–patient mismatch were implanted with a bioprosthetic aortic valve.

**Table 3:** Characteristics of the surgical procedure

Intraoperative data	Non PPM (*n* = 731)	PPM(138)	*P* value
CPB time	83(69–94)	83(69–93)	0.98
Cross-clamp time	51(42–64)	50(42–60)	0.33
Bioprosthesis	124(17.0%)	87(63.1%)	<0.001
Concomitant procedure			
Mitral valve procedure	397(54.3%)	77(56.1%)	0.78
Tricuspid valve procedure	96(13.1%)	17(12.3%)	0.79
CABG	14(1.9%)	8(6.1%)	0.015

*PPM* prosthesis-patient mismatch; *CPB* cardiopulmonary bypass; *CABG* coronary artery bypass grafting

### Factors affecting prosthesis-patient mismatch

According to a multivariate logistic regression analysis including all preoperative and intraoperative variables, patients with PPM had a higher incidence of female gender (*P* < 0.001; OR = 0.307; 95% CI, 0.19–0.486), a higher incidence of AS (*P* = 0.014; OR = 1.725; 95% CI, 1.118–2.663), higher body mass index (BMI) (*P* < 0.001; OR = 1.092; 95% CI, 1.029–1.160), and more frequently received a bioprosthesis (*P* < 0.001; OR = 13.907; 95% CI, 8.703–22.222) than those without a mismatch (Table 4).

**Table 4:** Logistic regression model for prosthesis-patient mismatch

Variables	Mean or %	OR	95% CI	*P*-Values
Female	48.1%	0.307	0.194–0.486	<0.001
AS	56.6%	1.725	1.118–2.663	0.014
Bioprosthesis	24.3%	13.907	8.703–22.222	<0.001
BMI	29.6	1.092	1.029–1.160	<0.001

*OR* odd ratio; *CI* confidence interval; *AS* aortic stenosis; *BMI* body mass index

### Major postoperative complications

There were no significant differences between the two groups regarding most of the postoperative patient data and complications, including perioperative blood transfusion, ventilation time, reintubation, duration of first time in ICU, reentering ICU, chest tube output, reoperation, sternal wound infection, postop stroke, postop renal failure, persistent atrial fibrillation (AF), multi organ failure, and length of hospital stay (*p* > 0.05). However, all-cause postoperative 30-day mortality was higher in the PPM group than in the non-PPM group (2.1% vs 0.5%, *p* = 0.050).

### Factors affecting postoperative mortality

Seven patients (0.8%) died during the postoperative hospitalization period, three (2.1%) with and four (0.5%) without PPM (*p* = 0.050). Five deaths were cardiac related; the other two patients (both without PPM) died due to severe infection. No patient died intraoperatively or within 30 days post discharge.

A binary multivariate logistic regression model was constructed to analyze factors relating to all-cause death (Table 5). The results of the analysis showed that patients with a smoking history (*P* = 0.001; OR = 44.780; 95% CI, 4.303–466.014) and a lower preoperative left ventricular ejection fraction (LVEF) (*P* = 0.004; OR = 0.884; 95% CI, 0.813–0.961) had a higher incidence of postoperative death. More strikingly, prosthesis–patient mismatch was found to be associated with global or cardiac early mortality (*P* = 0.005; OR = 16.493; 95% CI, 2.306–117.950).

**Table 5:** Logistic regression model for postoperative 30-day global mortality

Variables	Mean or %	OR	95% CI	*P*-values
Smoking history	17.5%	44.780	4.303–466.014	0.001
PPM	15.9%	16.493	2.306–117.950	0.005
Preoperative LVEF	61.33	0.884	0.813–0.961	0.004

*OR* odd ratio; *CI* confidence interval; *PPM* prosthesis-patient mismatch; *LVEF* left ventricular ejection fraction

## Discussion

Based on the aforementioned definitions, the incidence of PPM in our single-centered cohort was 15.9%, and only 0.5% of the cases met the criteria for severe PPM. Although highly variable, PPM rates in most of the literature were higher than 20% [8]. The low rate of PPM in this study might be related to the particular physical condition of East Asians. First, East Asian population has considerably smaller body surface area than western populations. Also, the patients in this district are generally younger due to higher incidence of rheumatic causes, demanding more mechanical prosthesis, which may explain the lower incidence of PPM. A similar low rate of PPM has been reported by Japanese and Indian scientists [9, 10].

### Predictors of PPM

We performed logistic regression analysis and found AS to be an independent risk factor for PPM. The reason why PPM occurs more frequently in patients with AS than aortic regurgitation is that patients with a stenotic native aortic valve tend to have smaller valvular annuli. It is also one of the reasons why older people, observed more frequently with calcified AS, had a higher incidence of mismatch. Another reason is that older patients, especially those aged over 65, were implanted with a bioprosthetic valve. Mechanical valves, compared to stented bioprostheses, have a more favorable relationship between the external diameter and the EOA [11], thus reducing the incidence of PPM. Diabetes and atherosclerosis may also be related to AS and old age, indirectly influencing the rate of PPM.

We also found that there were more female patients in the PPM group (59% female and 41% male), consistent with other studies [12]. The study showed that women had smaller aortic root diameters than men [13], making them more likely to be implanted with a small sized valve prosthesis. This may explain why there were more smokers in the non-PPM group of our study. Our study also showed that PPM was associated with higher BMI. Therefore, weight control should be urged in obese patients to minimize the adverse effects of PPM.

### Effect of PPM on early mortality

There were no obvious differences regarding early postoperative patient data and complications between the two groups. However, logistic regression analysis showed that PPM was an independent factor predicting postoperative 30-day all-cause mortality, along with smoking history and low preoperative LVEF. The high mortality may be associated with the increased hemodynamic burden imposed by PPM. Further, the combination of poor ventricular function and moderate to severe PPM may dramatically increase mortality risk [14].

### Clinical implication and prevention

Some studies have shown that PPM affects long- and short-term postoperative morbidity and mortality [14, 15], whereas other studies have proven that even severe PPM does not increase the incidence of adverse outcomes [4, 16].

Aortic root enlargement surgery is an alternative procedure to avoid PPM. However, preventative root enlargement surgery may be related to prolonged off-pump time and increased risk [17, 18]. Therefore, considering the relatively low rate of PPM in China, we did not take aggressive root enlargement as a routine procedure in patients with a risk of mismatch. However, the implantation of newer-generation biological or mechanical prostheses with or without aortic annular enlargement should be considered according to the characteristics of the patient and the risk-benefit ratio for carrying out a particular procedure in an individual patient.

Out data have shown that although PPM does not affect the incidence of postoperative complications, all-cause early mortality of the patients did increase. Therefore, a randomized controlled trial will be carried out next to evaluate the effect of root enlargement strategies in selected young patients.

The results of this retrospective study have updated our understanding of PPM and encourage a positive view of preventative root enlargement strategies in patients with PPM.

### Limitations of the study

This was a retrospective study; the analysis, therefore, has inherent disadvantages. The recorded differences in patient outcomes could originate from smaller recorded or unrecorded differences between the two groups.

EOA was predicted by reference tables, which may not reflect the actual *in vivo* values of the EOAi. The effect of PPM on long-term outcomes and survival was not studied. Moreover, this was a single-centered analysis, thus the extrapolation of the results should be treated cautiously. However the sample size is acceptable. Comparing to a multi-centered study, our single-center analysis have the advantage of minimizing the deviation in patient surgical outcomes caused by uneven surgical procedures, varied skills of surgeons and different myocardial protection strategies.

Nevertheless, a randomized prospective multi-centered clinical trial is needed for further evaluation of the effect of PPM on longer term hemodynamic function, left ventricular function, and overall patient outcomes.

## Conclusions

This study demonstrates that female gender, aortic stenosis, bioprosthesis, and high BMI are risk factors for PPM in patients undergoing AVR. PPM is associated with a higher short-term mortality after AVR in China.

## Abbreviations

AF: Atrial fibrillation
AS: Aortic stenosis
AVR: Aortic valve replacement
BMI: Body mass index
BSA: Body surface are
CABG: Coronary artery bypass grafting
CI: Confidence interval
CPB: Cardiopulmonary bypass
EOA: Effective orifice area
EOAi: Effective orifice area index
ICU: Intensive care unit
LVEF: Left ventricular ejection fracture
NYHA: New York Heart Association
OR: Odd ratio
PPM: Prosthesis-patient mismatch
